# Emotion-Aware Scene Adaptation: A Bandwidth-Efficient Approach for Generating Animated Shorts

**DOI:** 10.3390/s24051660

**Published:** 2024-03-04

**Authors:** Yi Yang, Hao Feng, Yiming Cheng, Zhu Han

**Affiliations:** 1 Tsinghua National Laboratory for Information Science and Technology, Department of Electronic Engineering, Tsinghua University, Beijing 100084, China; 2 School of Software, Tsinghua University, Beijing 100084, China; 3 Department of Electrical and Computer Engineering, University of Houston, Houston, TX 77004, USA

**Keywords:** semantic communication, large-scale generative models, reinforcement learning, high compression rates, metaverse

## Abstract

Semantic communication technology in the 6G wireless system focuses on semantic extraction in communication, that is, only the inherent meaning of the intention in the information. Existing technologies still have challenges in extracting emotional perception in the information, high compression rates, and privacy leakage due to knowledge sharing in communication. Large-scale generative-model technology could rapidly generate multimodal information according to user requirements. This paper proposes an approach that leverages large-scale generative models to create animated short films that are semantically and emotionally similar to real scenes and characters. The visual content of the data source is converted into text expression through semantic understanding technology; emotional clues from the data source media are added to the text form through reinforcement learning technology; and finally, a large-scale generative model is used to generate visual media, which is consistent with the semantics of the data source. This paper develops a semantic communication process with distinct modules and assesses the enhancements garnered from incorporating an emotion enhancement module. This approach facilitates the expedited generation of broad media forms and volumes according to the user’s intention, thereby enabling the creation of generated multimodal media within applications in the metaverse and in intelligent driving systems.

## 1. Bridging Human Perception and Multimodal Content Generation

As the commercial scale of content generation technologies such as ChatGPT continues to expand, the research on AI content generation models is widely conducted among academic researchers in artificial intelligence and related fields. The emergence of large-scale generation models represented by ChatGPT and Stable Diffusion represents the birth of artificial intelligence-driven content generation methods. Large language models (LLMs) represented by ChatGPT have been used to automate customer service and facilitate human–machine conversations such as chatbots or virtual text assistants. Moreover, there are more and more intelligent applications that strive to be integrated into this most widely used intelligent interaction platform by using text-, voice-, or image-based interactive functions (using ChatGPT as the entrance). Today, large-scale generative models are being continuously and innovatively applied in medicine, law, education, and other disciplines and are increasingly integrated into more industrial and commercial fields [1,2,3,4,5,6].

As the technology of large-scale generative models continues to develop, there are more and more interactively generated contents that utilize natural text as the basic medium for content preservation and dissemination. Language is an important carrier for human beings to communicate information with each other. Multimodal information (including text, speech, images, videos, etc.) generated using large-scale generative models greatly expands the scope and quantity of existing media. This enhancement not only enriches the diversity of expression forms but also significantly lowers the user threshold [7,8,9,10].

The metaverse is often used to describe the future of the internet, consisting of a persistent, shared, 3D virtual space linked to a sentient virtual universe [11,12,13,14]. One of the key technologies of the Metaverse is digital twins, that means, digital replicas of a large number of physical environments in the Metaverse. Digital twins are critical to creating immersive experiences in the metaverse, as they allow static and dynamic objects to be replicated and interacted with in virtual environments. The metaverse is characterized by immersion, interactivity, and persistence. These three characteristics require the support of immersive media, tactile internet, and wireless communication technologies, that is, integrating real and virtual domains exchanging multimedia data and natural signals. Metaverse has strong demands for quality for immersive experiences, which requires the wireless network it uses to ensure high levels of key performance indicators such as low latency and high throughput. In order to ensure the continuous increase of diverse intelligent services and machine-to-machine communications, wireless networks in the metaverse scenario may have to work on extracting useful communication semantics.

Among the much-discussed metaverse and 6G wireless system technologies, industry members and researchers have proposed deeply immersive experiences, which require a significant increase in data transmission volume, requiring data rates of several gigabits per second. This situation requires the use of holographic-type communication (HTC), which is known for being data-intensive; it includes not only holograms but also a range of multisensory media. It is easy to imagine that this technology requires considerable bandwidth in terms of data complexity and data volume. In order to cope with these challenges, it is necessary to consider changing the method and format of the transmitted content. In order to achieve efficient transmission in communications, it is necessary to perform advanced data compression optimization without affecting the quality of experience as much as possible, with the purpose of converting data into a more compact form. By doing so, the burden can be alleviated on network bandwidth and storage, making it feasible to deliver these rich, multisensory experiences even over limited bandwidth. This transformation is not just a technical necessity but also a strategic imperative to ensure the scalability and accessibility of these technologies. For example, point cloud video needs to be processed during rendering for holograms. Therefore, the substantial increase in data throughput causes network congestion and storage capacity overload, posing challenges to existing multimedia technologies. These current situations will inevitably affect the quality of user experience [15]. If point cloud data, which need to be processed during rendering for holograms, are compressed or transformed into a more efficient format for transmission and then decompressed at the receiving end, they can substantially decrease the necessary bandwidth. The purpose of this approach is not just to reduce data size; it is about smartly managing the data to maintain the integrity and quality of the immersive experience while optimizing the data transmission process.

In addition, the use of semantic communication in 6G communication technology is inevitably challenged by privacy leaks. 6G communications need to consider the trade-off between data utility and knowledge sharing. These characteristics will damage the reliability of 6G communication and expose users to privacy risks [16,17,18]. Therefore, semantic communication in 6G communication technology also requires more research and exploration into privacy protection. The main starting points may include optimizing data usage and maintaining privacy standards.

This paper proposes an approach that leverages large-scale generative models to create animated short films that are semantically and emotionally similar to real-life scenes and characters. The data sources for this approach are visual representations from real-world scenes, such as video interviews with specific people. The first step of this method uses semantic understanding technology to convert the visual content of the data source into a text expression. This step is to ensure that the main semantics of the data source media are retained while converting it into a highly compressed text form for transmission. Emotional cues from the data source media are then added to the textual form through reinforcement learning techniques. In the next step, these enhanced texts are used to generate semantically consistent visual media, especially in the form of animated short films. This stage is mainly accomplished using large-scale generative-model generation techniques. The advantages of the proposed approach are manifold. Initially, this method is beneficial to high compression rates at the semantic level, which facilitate efficient communication and storage of data; secondly, this method ensures the preservation of effective semantics of the original media (such as character interview videos), including emotional perception, which is used to achieve the goal of maintaining the emotional context of the original media content. Finally, this approach enhances privacy protection as it allows sensitive or personal scenes to be depicted in a more abstract animation format, thus protecting tuser identity and privacy to the greatest extent.

At the same time, within academic research on large-scale generative models, there is no consensus on the performance metrics used to evaluate these models. This article compares and analyzes the efficacy of these models in conveying emotional expression through generated content by examining the quality of emotional expression embodied in the final visually animated short films produced with different textual prompts. Such analysis helps us to understand and evaluate the difference in emotional depth that large-scale generative models can achieve.

In fact, our proposed system for generating animated visual content directly from real scenes has a wide range of applications in various fields. For example, it enables users to easily convert their favorite real-world scenes into animated sequences in the metaverse, which can greatly enhance the digital content available in the metaverse and lower the technical threshold for users to participate in and interact with this virtual space. Another example is intelligent assisted transportation. Using this model can assist drivers in easily understanding traffic information in areas with visual blind spots, allowing them to make timely and informed decisions to avoid traffic accidents. Finally, in the field of real-time video broadcasting and video conferencing, our model helps generate emotionally differentiated animated expressions, an approach that not only makes communication more vivid but also enriches the overall quality of the interaction.

## 2. Introduction

As communication technology continues to develop, 5G communication is a transition platform for artificial intelligence-driven communication methods. In 5G communications, semantic communication focuses on the intrinsic meaning of information rather than just accurately transmitting data at the digital level. Semantic communication can be considered as an intelligent agent between the source of information and the destination of transmission, at the same time, artificial intelligence algorithms are used to interact with the outside world. It can be argued that communication technology will place greater emphasis on context awareness and meaning centering, especially for the understanding and interpretation of information [19,20,21].

Semantic communication embodies the major change in the communication technical paradigm, that is, from the traditional signal transmission to the meaningful data and information transmission [22]. The adoption of deep learning technology in semantic communication, which is widely implanted in research and application fields such as NLP (Natural Language Processing) or CV (computer vision), will conduce to the promotion of semantic communication capabilities. At the same time, semantic encoders and decoders are crucial for information processing and transmission at the semantic level. In semantic communication, it is necessary to quantify the content of semantic information, thereby measuring the importance and quantity of semantic information. This measurement is generally achieved by using the definition of semantic entropy based on logical probability and the application of membership in fuzzy systems. The evaluation of semantic communication effectiveness adopts unique performance metrics to evaluate the quality of semantic communication, including the BLEU [23] score of text and other metrics such as PESQ and FDSD.

As mentioned above, interdisciplinary research combining deep learning and semantic communication is increasingly emerging. The advantage is that the computing power of deep learning and the understanding of semantic information can complement each other. Three core issues inherent in the intersection of deep learning and semantic communication include intrinsic meaning in bit data, semantic error quantification, and joint encoding. In end-to-end physical-layer communication methods, deep learning technology has shown superior performance to traditional systems, especially in terms of bit error rate (BER) indicators [24]. A typical example is the DeepSC system, which integrates semantic and channel encoders and decoders and adopts the transformer model and deep transfer learning to adapt to various communication scenarios. DeepSC’s loss function is based on sentence similarity and mutual information, where sentence similarity is defined as an indicator to evaluate semantic-level performance. The advantage of DeepSC is that the DeepSC system has good efficiency even at a low signal-to-noise ratio (SNR). Specifically, DeepSC extracts semantic information from text data to control channel noise and semantic distortion, so it can demonstrate good robustness in different communication environments.

Semantic extraction and propagation in communication systems are used to emphasize the importance of semantics/intent in the information transfer process, which is beneficial to enhancing the service quality and potential capabilities of 6G networks and metaverse scenarios [25,26,27]. One example is semantic communication systems in autonomous driving: the point is to extract and interpret hidden meanings from multimodal data (which can be real-time data from multiple sensors, such as radar or cameras), improving the efficiency of autonomous vehicles and Intelligent capabilities.

Currently, this type of intelligent communication mainly uses complex deep learning algorithms and neural network architectures, and these methods are usually specialized for certain types of data and therefore lack the ability to manage discrete constraints. Traditionally, Shannon-based theoretical frameworks consider the semantic dimensions of communication to be independent of specific technological frameworks. However, the emergence of 6G wireless communication technology has rekindled people’s interest in semantic communication technology. The researchers propose a so-called “semantic signature”, which employs a hash-based semantic extraction process, leveraging supervised learning techniques to generate unique, one-time, hash-based signatures. This strategy helps effectively manage the dynamic nature of semantic communications, ensuring efficiency, security, and reduced latency in the process. However, achieving reliable semantic communication in 6G communications is still a challenging task.

The technological capabilities of 6G, which are attracting increasing attention, must surpass 5G, which requires greater attention to the role of semantic communication, especially in supporting real-time decision-making applications. In order to overcome the inherent inefficiency problem of traditional “sampling and then compression”, semantic compressed sensing technology has been researched and applied for a long time, which could improve data processing efficiency and reduce network traffic. For example, sparse sampling of source data needs to be exploited in intelligent transportation systems to facilitate timely semantic perception and transmission, especially in emergency situations, such as pedestrians suddenly appearing. Furthermore, semantic communication technology integrates semantic-based indicators into the communication process, emphasizing the value-of-information (VoI) concept [28]. This concept is increasingly important in urban traffic environments, in vehicle-to-everything (V2X) collaboration, and in the field of health-related wearable devices, for example, in emergency situations where only critical data flows are considered.

There is another practical application problem in the use of 6G technology, namely, privacy leakage. A large amount of personal information and sensitive data are constantly transmitted and stored on mobile internet and IoT devices, leading to personal information leakage or unauthorized data tampering. The purpose of privacy protection technology is to prevent misuse of data or unauthorized access. Semantic communication emphasizes the importance of data content rather than pure bit-level transmission. Therefore, adopting semantic communication is effective in preventing misuse of data or unauthorized access [29]. By leveraging semantic communication technology, it is not only possible to identify the context in which data are used but also to process and transmit only relevant and necessary data. By understanding the semantics of data, semantic communication systems can intelligently decide which data to store or discard. By using semantic communication technology, the amount of sensitive data that edge nodes need to retain can be reduced and the storage requirements for edge nodes can be reduced. Not only that, semantic communication can also use technologies such as federated learning, differential privacy, or homomorphic encryption to further enhance privacy protection capabilities.

Large-scale generative models are reshaping many kind of fields, such as data processing, artificial intelligence, and human-computer interaction. These models are capable of generating a variety of content types (numeric values, text, images, audio, etc.) with levels of fidelity and complexity comparable to those generated by humans [1,30].

Under the dual requirements of efficient communication and massive data transmission, the communication industry needs a semantic communication method that can accurately extract the meaning or intention of the message. As mentioned above, compared with traditional communication methods that focus on the accurate transmission of bits, semantic communication emphasizes the understanding and interpretation of the meaning or intention of the message. It also helps to deal with bandwidth issues in massive data transmission scenarios.Large-scale generative models hold promise for meeting this requirement. Their model comes from training on massive data sets, so the model can distinguish the differences in context and semantics carried in the data source information. It can therefore be argued that the next interesting convergence of large-scale generative models with industrial applications is semantic communication. Therefore, in semantic communication, large-scale generative models can be leveraged to ensure that the essence of the message is conveyed and understood even if the exact words or syntax cannot be transmitted due to bandwidth limitations or transmission errors. In addition, large-scale generative models can adapt to different communication environments and user preferences, enhancing the personalization and effectiveness of the communication process. For example, in scenarios where a message needs to be compressed for transmission, a generative model can reconstruct the message to use fewer bits while retaining its core meaning. Additionally, in a world increasingly reliant on wireless communications, large-scale generative models can help reduce bandwidth burdens. In particular, in scenarios where bandwidth resources are scarce, communication efficiency can be greatly improved by focusing on semantic content rather than raw data.

So far, the purpose of combining large-scale generative models with semantic communication technologies has been to ensure the transmission of semantically important content and remove redundant data, thereby improving bandwidth efficiency. In other words, during the transmission process, the total amount of data transmitted should be minimized and the core information should be fully conveyed. The achievement of this dual goal therefore relies on evaluating the semantic importance of data frames while effectively managing the known power distribution. Compared with common semantic communication strategies, the deep joint source-channel coding (Deep-JSCC) proposed by the researchers is well compatible with current communication infrastructure [31]. It can be seamlessly integrated into existing systems by adding a cross-layer manager.

Researchers have also noticed that large-scale generative models can be used to achieve data compression. In particular, large-scale language models (e.g., GPT-4) have good practical value in text content compression. In such explorations, a systematic comparison of the compression ratios and reconstruction losses of these models with those achieved by traditional compression methods is requested [32]. Preliminary results show that while GPT-4 may not meet the standards for lossless compression, it performs well in maintaining semantic consistency. By leveraging advanced generative models such as GPT-4 for data compression prior to transmission, the amount of data transmitted can be effectively reduced, especially in the context of textual content, thereby reducing bandwidth requirements.The cosine similarity measure is used in the comparative analysis methodology section, which is a scale used to quantitatively evaluate the retention of semantic content during compression.

In addition, when adopting large-scale generative models for data compression, researchers have noticed the application of large language models (especially GPT-4) in text content compression. By performing a systematic comparison of the compression ratios and reconstruction losses of these models with those achieved by traditional compression methods (a key aspect of this comparative analysis is the implementation of the cosine similarity measure, a method used for the quantitative evaluation methods of preserving semantic content during compression) we learned that although GPT-4 may not meet the standards of lossless compression, it shows potential in maintaining semantic consistency, that is, GPT-4 can effectively compress and process information and extract and interpret data from more compact and simplified inputs.

Large-scale language models also show some potential in text-based sentiment analysis applications. ChatGPT, for example, shows average proficiency in such tasks, with an accuracy metric of 69.7 percent [33] in emotion perception. However, in the process of implementing semantic decoding using large-scale generative models, there are always errors or gaps in emotional expression. The reason may lie in the lack of human emotion in the textual expressions generated by semantic encoding, which seriously damages the authenticity of the subsequent visual media generated. The traditional standard metrics such as BLEU, ROUGE [34] and perplexity are mainly used to measure the effectiveness of models such as GPT-4. However, there is still a gap in developing evaluation metrics specifically for comparing the performance of large-scale generative models (e.g., GPT-4), especially human emotion representations.

To date, the application potential of combining large-scale generative models with semantic communication is limitless. For example, in the metaverse scene mentioned above, if semantic understanding is used to encode actual scenes and characters into text form, it only takes up a minimum of transmission bandwidth and can reconstruct virtual scenes and characters that are semantically consistent with the real world on the decoding end. This reconstruction is performed using a large generative model. Another example is the above-mentioned intelligent assisted-driving scenario. By using semantic communication to encode the real visual information of the driver’s blind spot area, it is decoded on the vehicle side to restore a virtual scene that is semantically consistent with the blind spot area. The large-scale generative models can be used when reconstructing information. This method not only alleviates the massive demand for bandwidth but also enhances privacy protection during communication. These advantages are likely to become a feature of data transmission in the 6G era. However, to achieve this goal, the best existing related techniques still suffer from some shortcomings, such as the lack of emotional expression in scenes and characters and the lack of standardized evaluation metrics to measure their performance.

We propose a bandwidth-efficient approach for semantic communication through emotion-aware scene adaptation on the encoding side and generating animated video short on the decoding side. By including emotional expressions in semantic communication and establishing standardized evaluation metrics for this purpose, this approach achieves enhanced semantic and emotional consistency between original video content and generated animated short representations.

Our solution is built around a three-step process. Firstly, the original video content needs to extract key frames and convert them into text description representations.This step is crucial for the desired bandwidth-efficient nature, since in this step the basic semantic and emotional content is conveyed in a very compact textual form, which significantly reduces the data required for transmission. At the same time, as mentioned above, in addition to reducing bandwidth consumption, this encoding method can also ideally protect privacy, because most privacy-related information expressions can be removed during the encoding process.

In the second step, the text description representations of the first step are obtained at the decoding end, and specific emotional text clues are added to them. This step serves the goal of preserving the emotional integrity of the original video, i.e., by embedding these emotional descriptors into semantic text description representations, the animated short film generated by the large-scale generative model is not only able to preserve the semantic context of the original video, but also can retain its emotional tone and nuance.

Finally, the consistency in terms of content and emotional expression between the generated animated short film and the input original video is defined and evaluated. As an evaluation metric, this consistency allows the model to be fine-tuned with the goal of achieving high fidelity while reproducing the semantics and emotional tone of the original video. Through this evaluation metric, in some specific application scenarios, the requirements for emotion retention and representation are met.

Our proposed bandwidth-efficient approach hopes to ensure that the transition from real-world scenes/characters to their digital counterparts is seamless and efficient, as well as semantically and emotionally consistent. In addition, the proposed metrics will also better evaluate the performance of large-scale generative models in terms of semantic and emotional expression, which further explores the potential of semantic communication combined with large-scale generative models.

## 3. Methods

**Figure 1:**
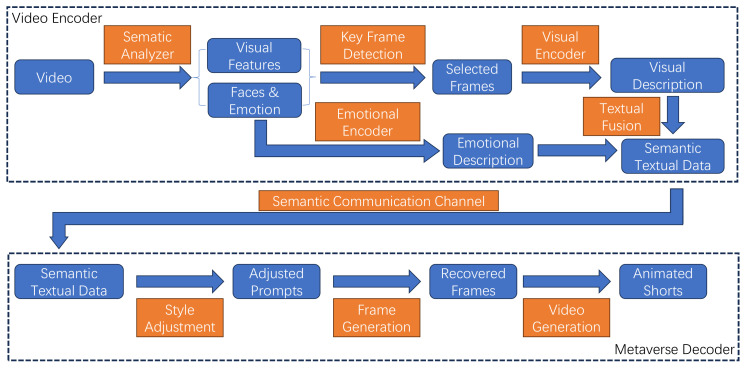
The architecture of the proposed method.

In this section, we detail the methodologies employed to transform video content into animated representations, with a focus on preserving both the semantic integrity and the emotional context of the original material. As shown in Figure 1, the approach is founded on a sophisticated encoding system. This system initiates with the meticulous acquisition of visual data, followed by their transformation into a semantically and emotionally enriched textual format before finally culminating in the generation of animated shorts. To achieve this, we utilize a semantic communication channel designed specifically for transmitting semantically converted textual data, rather than relying on the transmission of raw video footage or images extracted from the videos. This method significantly reduces the volume of data required for transmission, presenting a stark reduction in comparison to the volume of the original video data. Crucially, by integrating emotional descriptions directly into the semantic textual data, we ensure that the resulting animated shorts not only convey the original video content’s semantic meaning but also its emotional nuances. This integration allows for a nuanced reflection of the original video’s emotional undertones in the animated representations, ensuring a faithful and comprehensive translation of content and emotions.

Firstly, the input video is preprocessed, including reading video data, adjusting the video based on given resolution frame rate and other parameters, and removing noise. In the subsequent step (*Semantic Analysis*), visual features are extracted, and the faces appearing in the video are recognized and analyzed to obtain their representative emotions, laying the foundation for subsequent processing.

In order to avoid high computational costs and lengthy results, representative key frames in the video are identified, and only these frames need to be processed in subsequent conversions (*Key Frame Detection*). In this step, visual features of the video as well as facial and emotional information are used.

For the selected key frames, their image information is encoded and processed into textual descriptions (*Visual Encoder*). The emotional information of the previously recognized faces is also encoded and recorded in text form for each frame with facial information (*Emotional Encoder*). Through this method, the content contained in the video is expressed and converted into text format (visual description and emotinoal description).

A key step in the method is to integrate visual and emotional descriptions into a coherent semantic text narrative (*Textual Fusion*), using reinforcement learning methods to adjust how the two types of descriptive information should appear in the final fused text. Afterwards, the fused composite data with rich semantic information can be efficiently transmitted through semantic communication channels (*Semantic Communication Channel*).

After receiving the fused text description, it is necessary to generate animation frames from this semantic encoded data, and then generate animation short films. This includes adding style information related to animation to the text description (*Style Adjustment*), reconstructing corresponding animation video frames from the text (*Frame Generation*), and reconstructing the obtained animation video frames into an animation sequence that reflects the original content (*Video Generation*).

The methods outlined in this article can completely change the way we interact with multimedia content in virtual environments by transforming video content and being applicable to the metaverse environment.

### 3.1. Preprocessing and Semantic Analysis

After receiving the input raw video, in order to be applicable to subsequent conversion processes, preprocessing and semantic analysis are required. At this stage, input data is standardized and processed to address the issue of different input data formats, and different content obtained from semantic analysis is processed to address its inherent heterogeneity in format.

#### 3.1.1. Video Standardization

Due to the diversity in format, resolution, frame rate, and content of the input video, an adaptive scaling algorithm was adopted. This algorithm standardizes all input raw videos to predefined setting $R_{s}$, facilitating the use of a unified method for processing in subsequent feature extraction and analysis processes. The resolution normalization function can be mathematically expressed as

(1) $$R_{normalized}=f_{scale}\left(V_{input},R_{s}\right)$$

where $V_{input}$ is the input video and $f_{scale}$ denotes the scaling function that adjusts $R_{input}$ to match $R_{std}$.

#### 3.1.2. Noise Reduction

To counteract the noise prevalent in real-world videos, spatial filtering and temporal smoothing are implemented. The noise reduction process can be represented as

(2) $$V_{clean}=f_{noise\_reduction}\left(V_{raw},\alpha \right)$$

where $V_{raw}$ is the raw video frame, $V_{clean}$ is the noise-reduced frame, and α is a parameter controlling the intensity of the noise-reduction process. $f_{noise\_reduction}$ encapsulates the combination of spatial and temporal filtering techniques.

#### 3.1.3. Semantic Analysis and Feature Extraction

The cornerstone of our methodology, semantic analysis, involves extracting salient features carrying substantial semantic weight from the video frames as follows:

- **Visual feature extraction**: Convolutional neural networks (CNNs) are used to process image frames in food to extract key visual features, such as object content, environment, and image style information contained in the image. The feature extraction can be represented as
  (3) $$F_{visual}=CNN\left(V_{clean}\right)$$
  where $F_{visual}$ represents the extracted visual features from the noise-reduced video frame $V_{clean}$.
- **Facial and emotion recognition**: Facial recognition and emotion detection models recognize facial information (including the number and position of faces) in a given frame and analyze the facial expressions and emotional states on the recognized faces. The process can be formulated as
  (4) $$E_{emotion}=f_{emotion}\left(F_{facial}\right)$$
  where $E_{emotion}$ denotes the encoded emotional states and $F_{facial}$ represents the facial features extracted from the video frames.

#### 3.1.4. Data Homogenization

To prepare for key frame detection, we integrate the visual features extracted from key frames and the emotional data recognized in each frame into a unified data structure. This involves aligning two types of data and storing them in a fixed format for efficient processing in subsequent stages. Data homogenization can be mathematically represented as

(5) $$D_{homogenized}=f_{homogenize}\left(F_{visual},E_{emotion}\right)$$

where $D_{homogenized}$ is the unified data structure and $f_{homogenize}$ is the function that combines visual features $F_{visual}$ with emotional data $E_{emotion}$ into a coherent format. These homogenized data lay the foundation for the next stage of our method, ensuring consistency and efficiency in the processing process.

### 3.2. Key Frame Detection

Key frame detection is a crucial step in ensuring the selection of the most representative and emotionally significant moments in the video for further analysis. Our system uses change detection algorithms to detect frames that exhibit significant changes in scene, action, or emotional tone. The detection algorithm can be represented as

(6) $$KF=f_{detect}\left(\left\{D_{homogenized}\right\},\theta_{change}\right)$$

where KF is the set of key frames, $\left\{D_{homogenized}\right\}$ is the set of all features extracted from each frame of the input video, $\theta_{change}$ is the set of thresholds for changes in scene, action as well as emotional tone, and $f_{detect}$ represents the detection function.

### 3.3. Generating Textual Information

After processing, the input video will form textual content, greatly reducing the bandwidth required for information transmission.

#### 3.3.1. Visual Encoder and Emotional Encoder

Previously, visual feature information and facial and emotional information contained in each frame were obtained, and key frames in the original video were detected. But now the visual and emotional feature data is still in the form of feature vectors, which can be transformed into text information that is easy for humans to understand.

For the detected key frames, the visual information contained therein is encoded into text form for subsequent processing. This involves deep learning techniques that use image to text conversion, which can preserve the semantic information of the original image as much as possible during data compression.

For facial information and emotional information, they can be recorded in the form of text descriptions, making it easier to understand the character and emotional information in the scene during the subsequent restoration process.

#### 3.3.2. Textual Fusion

The textual fusion algorithm synthesizes visual and emotional text data, creating a coherent description that represents the semantic and emotional information of the original content. In this process, the emotional information of the key frame should be integrated into the visual description of this frame to obtain a complete semantic text description. Due to the various ways in which two pieces of text information can be fused, reinforcement learning methods can be applied, and the evaluation of the final generated results promotes a more reasonable result of text fusion.

The generated semantic text data serves as the blueprint for animation, encoding not only visual information but also characters and emotional information in the scene, making it easy to restore the original emotions in the generated animation.

### 3.4. Semantic Communication Channel

The semantic communication channel is a way for synthesized semantic text data to be transmitted to animation generation systems, emphasizing security and efficiency.

During the transmission process, the integrity and security of data transmission are crucial, and the efficiency of data transmission is also a key focus.

In order to achieve efficient data transmission, the fused text information is encoded and compressed, while preserving the information while reducing bandwidth usage. In order to protect data during transmission, ensure semantic integrity and confidentiality, secure transmission protocols are used during the transmission process.

### 3.5. Adjustments for Animation Generation

Before generating the final animation content, the transmitted text data undergoes a series of adjustments.

Due to the different animation styles of the input video and output, the fused semantic text needs to be adjusted to adapt to the expected animation style and tone, ensuring that the final generated result is consistent with the target requirements.

In addition, in order to ensure the compatibility between text information and animation generation algorithms, fine-tuning data is involved to lay the foundation for creating frame sequences in animation.

### 3.6. Frame Generation and Animated-Shorts Production

In this stage, semantic text is transformed into animated short films that can summarize the essence of the original video content. The generated animated short film and the original video remain consistent in content and emotion.

#### 3.6.1. Semantic-to-Visual Frame Generation

By using a text to image generation model based on deep learning, images can be synthesized from semantically rich text descriptions. In this process, it is necessary to convert the detailed description in text format into visual elements to ensure that each frame accurately presents the expected scene and restores the original content of the input video. During the generation process, attention should be paid to the consistency between frames to avoid too many drastic changes and ensure the coherence of the generated animation sequence.

#### 3.6.2. Animated Short Film Production

Creating animated short films is the final step in the visual transformation process.

Based on synthesized animation frames, it is necessary to serialize and animate them, use editing tools to fill the gaps between frames, and create a vivid and engaging short film. This includes adding motion, transitions, and other visual effects to make the story more vivid. And it is necessary to integrate audio elements such as dialogue, music, and sound effects into the animation to enhance the overall sensory experience and emotional impact of the short film.

The animated short films generated in this way not only visually attract the audience, but also convey the rich emotions and narrative depth of the original content.

### 3.7. Metaverse Decoder Integration

The method of generating animated short films can ultimately be integrated into the metaverse environment, making it easier to generate animated short films on demand in the metaverse and experience the resulting short films.

#### 3.7.1. Adaptation for Metaverse Platforms

We need to adjust the format of the animation content and perform special encoding to match the technical and experiential requirements of the metaverse platform.

In terms of format and resolution, optimization is needed to ensure that the animation is presented in a format compatible with the metaverse platform, and to optimize resolution and frame rate to meet the standards of virtual reality environments.

Embedding interactive features into animation content allows users to participate in the story plot and characters in a more immersive way.

#### 3.7.2. User Experience and Engagement

Our process of integrating animation into the metaverse focuses on enhancing user experience and engagement. Animation is placed in the metaverse as an immersive narrative element, providing users with a new way to experience narrative and connect content. We study and integrate user interaction dynamics to ensure that animation content is not only visually appealing, but also positively responsive to user input and behavior within the metaverse. Through these methods, we have achieved seamless integration of emotionally rich and semantically profound animation content with the metaverse, enhancing the narrative depth and interactive engagement of virtual experiences.

## 4. Experiments

In this section, we succinctly summarize the overall framework of the experiment and construction of the model. In Section 4.1, we briefly delineate the description of the datasets utilized in the experiment and the process of dataset preprocessing.

### 4.1. Dataset and Preprocess

- **eRisk 2017:** In the process of communication, we specifically focused on depression, which is a strong emotional state during the emotional detection phase. For this purpose, we utilized the dataset from the CLEF 2017 public task, which is designed for the early detection of depression in users. This original dataset, sourced from Reddit, classifies subjects into two categories: “depressed” and “control”, and contains extensive sequences of user posts. The ground truth of the data originated from manual annotations by the dataset’s creators. Initially, personal declarations of confirmed depression diagnoses among users were searched. Subsequently, these posts were verified and labeled through a manual review process. Posts without explicit indications of a diagnosis were not included in the depressed group. Therefore, the dataset generally possesses high reliability, but it still may contain some noise due to the absence of explicit diagnostic statements in certain posts. It is important to note that the original dataset had a substantial imbalance between the positive and negative samples, with 137 positive and 752 negative samples in the training and test sets. Therefore, in the data preprocessing stage, we first balanced these samples by downsampling the negative ones, resulting in an equal number of 137 samples in both categories. Since the dataset is presented in the form of individual user post sequences, it aligns well with the process of communication.The final dataset, after undergoing a series of processes for cleansing missing values and invalid characters, was converted into a more manageable CSV format. The utilization of the dataset adheres to a set of guidelines based on the data usage agreement signed with the dataset’s authors.
- **SWDD:** In our comparative analysis, we selected a dataset comprising sequences of user-posted content sourced from Weibo. This dataset encompasses a total of 3711 positive samples and 19,526 negative samples.The dataset, being primarily in Chinese, also presents the challenge of a significant imbalance between positive and negative samples. Moreover, the Weibo dataset contains numerous extraneous elements such as forwarded tweets and advertisements. To address these issues, we initially undertook a preprocessing step. This included a random downsampling of negative samples to balance the number of positive and negative samples at 3711 each. Furthermore, we employed the dataset author’s cleansing tools to eliminate irrelevant content, ensuring that only user-posted content was retained for analysis. It is noteworthy that this dataset was manually annotated by professional medical students, thereby ensuring a high degree of authenticity and reliability in the data. In the experiment, SWDD is included to compare and evaluate the performance of the emotion enhancement module. In subsequent applications involving Chinese language contexts, further optimizations will be implemented.
- **VideoEmotion-8:** In an effort to closely mimic the multifaceted emotional data sources present in real-world communication scenarios, we selected the VideoEmotion-8 dataset for our test video dataset. This dataset comprises 1101 videos sourced from YouTube and Flickr, averaging 107 s in duration, and is primarily focused on human emotion recognition. Building upon this foundation, we randomly selected five videos from each of the eight distinct emotional labels and two different video sources, forming a preprocessed dataset. Consequently, our dataset encompassed 80 videos, ensuring a diverse and balanced representation of emotions. This rich dataset provided a varied and extensive range of emotional expressions, thereby enhancing the efficacy and accuracy of our analysis and recognition models.

### 4.2. Evaluation Metric

- **PAD emotional classification:** In our experiment, we employed a multistage emotion recognition pretrained model named EmoCap, which incorporates content recognition using InceptionV3, to obtain a PAD vector for key frame classification in videos. The pleasure–arousal–dominance (PAD) emotion scale is an instrumental framework for the empirical assessment of emotional states. Each dimension of the PAD scale represents the quantified levels of pleasure, arousal, and dominance dimensions, providing a unified metric for the embedding of emotions. In the experiment, we utilized the pretrained EmoCap to convert images into three-dimensional PAD vectors. Then, by considering the sign of each dimension of the PAD vectors, we mapped them into the emotional space. The specific mapping relationship can be referred to in Table 1.
- **Accuracy of depression recognition:** In our study, utilizing a Reddit dataset with ground-truth annotations, we employed three key metrics for the identification within text sequences: precision, recall, and the F1 score. We conducted extensive training on the eRisk 2017 and SWDD datasets. The details of this training process, along with the results, are comprehensively presented in Section 4.4. Given the emphasis on detecting depressive moods, the accurate identification of positive samples becomes paramount. Therefore, these three metrics were chosen as the primary indicators of our model’s training efficacy, particularly in the context of emphasizing the detection of depressive emotional states:
- **Coverage of key scene detection:** The key frames extracted from the video should satisfy two main criteria: the minimal computational cost and the maximum diversity.
  **Representativeness:** The $R(f_{i})$ of a frame is quantified by averaging the similarity measures across all other frames, where the similarity could include aspects like image embedding, text embedding, and the emotional expressiveness of persons within the frames.
  **Information richness:** The $I(f_{i})$ is captured by the entropy of the symbols in the frame, such as pixel intensities or colors.
  For a single frame $f_{i}$, the coverage index can be denoted as $M(f_{i})$, which combines representativeness and information richness in the following way:
  $$M\left(f_{i}\right)=\alpha \left(\frac{1}{N}\sum_{j=1}^{N}\mathrm{sim}\left(f_{i},f_{j}\right)\right)+\beta \left(-\sum_{k}p_{k}\log\left(p_{k}\right)\right)$$
  For multiple frames, the coverage index M(S) for a set of frames *S* is given by the maximum similarity sum (MSS) and the collective information richness:
  $$M\left(S\right)=\alpha \left(\mathrm{MSS}\left(S\right)\right)+\beta \left(-\sum_{k}p_{k}\log\left(p_{k}\right)\right)$$
  $$\mathrm{MSS}\left(S\right)=\sum_{f_{i}\in F}\max_{j}\mathrm{sim}\left(f_{i},f_{j}\right)$$
  Here, the goal is to maximize M(S), ensuring that the key frames are as representative and informative of the video content as possible.
- **Accuracy of end-to-end communication:** In our simulation of semantic communication, we enhanced the semantics of the transmitted content with emotional attributes. To evaluate the results of emotion detection, we conducted an end-to-end assessment of emotional congruence. This involved matching the emotional features of the content before and after semantic transmission. The matching rate obtained from this process serves as a metric to assess the impact of communication on emotional stability. This approach not only evaluates the effectiveness of the emotional enhancement but also gauges how well the transmitted semantics preserve the intended emotional context through the communication process.
- **Compression ratio:** In our approach, we transformed original video data into semantic data, enabling the reconstruction of key frames and their associated emotional information, especially in bandwidth-constrained scenarios. Consequently, the data compression ratio was chosen as a crucial metric for evaluating the communication process. Due to the substantial magnitude of the final compression ratio data, we adopted a logarithmic transformation of the compression ratios in our presentation. This approach enables a clearer and more intuitive visualization of the data.

### 4.3. Implementation Details

Due to the presence of multiple modules, special attention was needed to address potential environment conflicts between them. We will detail the specific environmental configurations in the source code to publish. The final experiment was operable on an Nvidia 2080 with 12 GB VRAM; however, due to hardware limitations, the model operated slowly, and many of the finer feature extraction tasks for images can only be further optimized with more powerful hardware in the future.

The eRisk 2017 dataset used in this experiment involves user privacy and potential other issues, so access to the dataset can be obtained by applying to the original authors. On the other hand, the SWDD is a publicly available dataset annotated by professional medical students. The construction and testing code for the various modules, along with additional resources, will be made available postpublication of the paper, subject to application.

### 4.4. Experiments Details

#### 4.4.1. Experiment Setup

In the context of this experimental study, we employed a sophisticated hardware configuration, notably featuring an Nvidia 2080 graphics card endowed with 12 GB of VRAM. This choice was influenced by a commitment to optimizing the utilization efficiency within real-world systems. Consequently, we opted for a stepwise parallel methodology over the conventional serial approach for the purpose of channel simulation. This strategic decision was informed by the imperative to effectively manage the operational capabilities of the system.

Regarding the organization and accessibility of experimental data, all pertinent information was meticulously stored and cataloged in CSV format. This approach not only facilitates efficient segmentation of tasks but also substantially enhances the effectiveness of data processing and retrieval operations. The experimental setup was intentionally designed with modularity in mind, allowing for each component to be independently decoupled and fine-tuned via segmented simulation techniques.

A critical component of our experimental design was the oversight of connections between modules. We identified a significant risk of data loss during the transfer of identical objects across different modules. To mitigate this risk, we devised a specialized exit strategy, explicitly designed for such contingencies within the experimental paradigm.

The culmination of our experiment entailed a comprehensive and multifaceted testing regime. We executed an exhaustive comparison between our innovative method and the conventional benchmarks to assess the relative efficacy. Our testing protocol was extensive, encompassing various aspects of video data analysis, including the examination of individual video frames, key frames, and an all-encompassing dataset of the complete video sequence. This rigorous testing framework ensures that our data collection is not only broad-ranging but also aligns with the objective of garnering objective and thorough experimental insights.

#### 4.4.2. Depression Identification Module

Based on the refined eRisk 2017 and SWDD datasets, where extraneous information was removed and a balance between positive and negative samples was achieved, we conducted semantic detection and enhancement of depressive emotions. Our study involved comparing an array of model combinations, as detailed in Table 2. Given that our task centered on using sequences of individual user posts on social media as the dataset, aggregating all posts of a single user into one sample provided a more objective and comprehensive reflection of the semantic emotional characteristics. This approach effectively minimized the potential confounding variables that might arise from using single posts as the unit of data.

Results of experiments on this module are enumerated in Table 2, incorporating a variety of vectorization and model-fitting methods. A unique aspect of this task is the need to consider the characteristics of both individual sentences and the sequence of user posts. Consequently, our methodology included several vectorization techniques: some at the word level, such as GloVe and FastText, and others at the sentence level, like BERT and its optimized variant, MentalRoberta.

In terms of synthesizing these vectorized data into a comprehensive representation, we employed different approaches, including simple averaging and more sophisticated methods like weighted TF-IDF, which factors in term frequency.

For the model-fitting aspect, given that the task closely aligns with binary classification, we primarily utilized logistic regression. However, we also explored other regression methods, such as XGBoost 2.0.3, to evaluate their efficacy in this context.

We conducted a final evaluation of the model on three quantified metrics, with a particular emphasis on the identification of positive samples. Among the methods tested, the TF-IDFh + XGBoost and MentalRoberta + LR approaches demonstrated commendable performance in terms of the composite F1 score and recall values. However, it was observed in our experiments that models based on BERT exhibited a significant drawback in terms of slower computational speed. Despite the absence of specialized tools for the Chinese dataset, we decided to implement the MentalRoberta + LR method, which showed optimal performance on the Chinese dataset as the depression emotion identification module. This decision was made in anticipation of future tasks that may require specific adaptation to Chinese linguistic contexts, thereby laying the groundwork for more in-depth Chinese language-based tasks.

#### 4.4.3. Key Frame Extraction Module

Striking a balance between minimal computational demands and maximal diversity, we extracted affective key frames for segmentation of the video, which were then relayed to subsequent modules for further processing.

#### 4.4.4. Semantic Encoding Module

The most critical component of semantic communication is the transformation of original multimodal data, such as raw video or image data, into meaningful semantic encoding, as opposed to traditional encoding methods. This module must also prioritize being sufficiently lightweight and fast while minimizing the need for dense computations. In our experiment, we utilized CLIP to convert original key frames into a series of descriptive prompts. This module focuses primarily on content rather than emotional information. Consequently, we later integrated emotional information through an emotion enhancement module.

#### 4.4.5. Semantic Decoding and Recovery Module

This module’s function is to reconstruct the original modal data from the semantic information received at the communication receiver end, which were transmitted from the sender. Due to constraints in computational power and processing speed, the primary focus is initially on the restoration of key frames after their transmission. Considering the cumulative computational demand generated by the entire system, it is imperative that the recovery model be as lightweight as possible. Therefore, we ultimately employed Stable Diffusion as the semantic decoding and recovery module.

#### 4.4.6. PAD Emotion Mapping Module

The primary function of this module is to convert the data from their original modality into PAD vectors. By processing the data through EmoCap, vectors are obtained for each of the three dimensions of PAD. In our experiment, by embedding the key frame images into the emotional space, we acquired three-dimensional vectors. This allows for a distance measurement in the emotional space between the original images and those reconstructed post-transmission through the channel. Additionally, the obtained PAD vectors can be mapped onto the emotional space, resulting in a hard classification into eight distinct emotional categories. The criteria for these classifications are detailed in Table 1. The emotional classifications obtained can also be utilized in subsequent semantic emotion enhancement modules.

#### 4.4.7. Semantic Emotion Enhancement Module

In the original semantic context, the focus is primarily on the content of the original image, with no additional emphasis on emotional information. Therefore, in this module, we incorporated the emotional recognition keywords generated by the previous module into the transmitted semantics to enhance the emotion in semantic context.

## 5. Results

We constructed a semantic communication process based on four modules and compared the improvements brought about by the integration of an emotion detection enhancement module, resulting in a series of statistical outcomes.

### 5.1. Recall in Semantic Communication Emotion Detection

**Figure 2:**
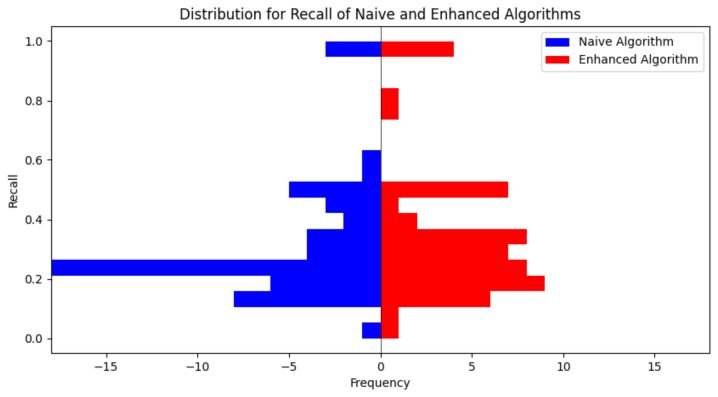
This graph compares the recall values for all images in each video with emotional enhancement (“Enhanced Algorithm”) and without emotional enhancement (“Naive Algorithm”) on the preprocessed dataset.

A critical aspect of our methodology is the deliberate integration of emotional information (the emotional enhancement). To quantitatively evaluate the influence of this integration on the quality of the generated animated shorts, we conduct an ablation study using the dataset VideoEmotion-8. The primary objective of this study is to systematically quantify the degree of enhancement in emotional consistency achieved by our approach, effectively demonstrating the benefits of incorporating emotional data into the transformation process. We compare our method (termed “Enhanced Algorithm”) with a comparable baseline method that does not incorporate emotional information (termed “Naive Algorithm”). The comparative results, illustrating the superiority of our approach over the baseline, are meticulously tabulated in Table 3. Furthermore, we present a detailed analysis of the distribution of recall values of all images of the videos for both methods in Figure 2, which encompasses two histograms corresponding to the two methods, respectively.

Despite limitations in dataset size and hardware resources, we achieved a significant average accuracy improvement of 3.19%, as shown in Table 3. The maximum increase in accuracy is approximately 11.7%. This demonstrates the effectiveness of the method to a certain extent.

Additionally, there is a slight improvement in the distribution of recall values, as depicted in Figure 2. In the two histograms in Figure 2, we iterate all the videos, calculate the recall value of all images for each video, then plot the histogram. We find that our approach (“Enhanced Algorithm”) tends to have higher recall values in the distribution. Therefore, emotional enhancement is useful for keep the emotional information of raw videos.

In the future, specialized enhancements based on dedicated datasets will be applied to the other seven emotions, which is expected to yield even greater improvements in subsequent tasks. Building upon the demonstrated feasibility, further optimization to enhance accuracy remains a future goal.

### 5.2. Compression Ratio of Transmitted Semantics Relative to Original Video

**Figure 3:**
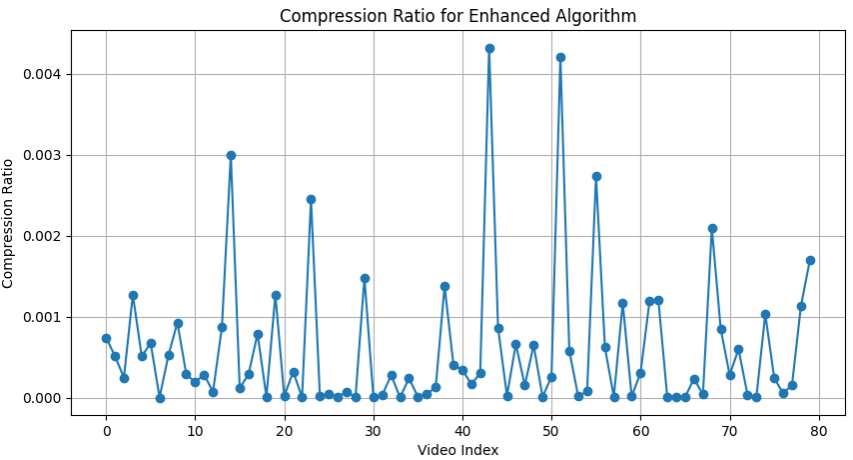
This graph depicts the compression ratio of the semantic information relative to the original video on the preprocessed dataset.

We define the “compression ratio” as the quotient obtained by dividing the size of the textual representation, intended for transmission, by the size of the raw video data. For example, if a video that is 100 MB in size has a textual representation that is only 0.2 MB, the compression ratio would be calculated as 0.2/100=0.002. Consequently, a diminished compression ratio directly correlates to a reduced requirement for bandwidth, signifying increased efficiency in data transmission. In Figure 3, we present the compression ratio performance on our preprocessed dataset. This graph plots the video index on the horizontal axis against the compression ratio on the vertical axis, showcasing how the efficiency of data compression varies from one video to another after applying our preprocessing techniques. Simultaneously, in Table 4, we depict the distribution of compression ratios. It is evident that the compression ratio is significant, averaging around 0.0005. This demonstrates that in scenarios with limited bandwidth, a sufficient compression ratio allows our semantic communication to transmit information effectively.

## 6. Conclusions

In conclusion, this paper introduces an innovative method leveraging large-scale generative models to create animated short films based on some input videos, which accurately reflect the semantics and emotions of original scenes and characters. By converting the visual content of data sources into semantic textual expressions, the bandwidth requirements can be significantly reduced because only the compact textual information rather than bulky original video data should be transmitted. By enriching these textual expressions with emotional cues via reinforcement learning technology, our methodology facilitates the generation of visual media that remain faithful to the original data source’s semantic content.

Furthermore, this research pioneers the development of a comprehensive semantic communication process, featuring distinct modules including an emotion enhancement module. The integration of this module not only contributes to the semantic richness of the media but also plays a crucial role in the transmission efficiency. Empirical evidence from our studies, particularly utilizing the third-party dataset eRisk 2017, confirms that the proposed approach successfully integrates emotional information and significantly reduces the bandwidth requirements for data transmission. This enhancement in both semantic integrity and bandwidth efficiency not only marks a significant advancement in our approach but also highlights its capacity to capture and convey crucial information, such as character emotions, in bandwidth-constrained environments. This capability ensures the creation of tailored animated shorts that resonate with the original video’s essence, making it particularly valuable for immersive experiences in the metaverse and other applications where bandwidth may be limited.

By bridging the gap between semantic understanding and emotional conveyance and by efficiently managing data transmission, this paper not only sets a new benchmark for the creation of multimodal media but also ensures the conveyance of critical emotional and semantic content in bandwidth-constrained environments. This advancement steers the future of media generation towards a horizon that is not only more expressive but also more efficient in bandwidth usage, paving the way for innovative applications in immersive experiences and beyond. Future work will explore extending this framework to encompass a broader spectrum of emotions and scenarios, further enhancing the adaptability and scope of semantic communication in next-generation networks.

## Figures and Tables

**Table 1:** PAD vector to emotion space mapping.

PAD Sign	Emotion
+P+A+D	Happy
− P−A−D	Bored
+P+A−D	Dependent
−P−A+D	Contemptuous
+P−A+D	Relaxed
−P+A−D	Anxious
+P−A−D	Gentle
−P+A+D	Hostile

**Table 2:** Comparison of different models on Reddit and Weibo datasets.

	eRisk 2017				SWDD		
	F1-Score	Precision	Recall		F1-Score	Precision	Recall
glove + LR	0.63158	0.60000	0.66667		-	-	-
FastText + LR	0.74194	0.65714	0.85185		0.65147	0.61871	0.68790
bert + LR	0.65144	0.61871	0.68790		0.91489	0.92275	0.90717
HAN + LR	0.655172	0.63333	0.67857		0.79570	0.75510	0.840909
LIWC + LR	0.69565	0.88889	0.57143		-	-	-
TF-IDF + XGBoost	0.73469	0.85714	0.64285		0.78483	0.80367	0.76685
MentalRoberta + LR	0.70588	0.78261	0.64286		0.91650	0.93132	0.90160

**Table 3:** Advancement in accuracy of using emotional enhancement compared to the baseline method without emotional enhancement. A positive number means improvement over the baseline.

Metric	Value
mean	0.031989
std	0.016137
min	−0.026315
25%	0.027017
50%	0.033750
75%	0.042941
max	0.117647

**Table 4:** Compression ratio statistics.

Metric	Value
count	80
mean	0.000599
std	0.000874
min	0.000002
25%	0.000037
50%	0.000280
75%	0.000804
max	0.004325

## Data Availability

Data are contained within the article.
